# A Family of Energetic Materials Based on 1,2,4-Oxadiazole and 1,2,5-Oxadiazole Backbones With Low Insensitivity and Good Detonation Performance

**DOI:** 10.3389/fchem.2019.00942

**Published:** 2020-02-20

**Authors:** Qi Xue, Fu-qiang Bi, Jun-lin Zhang, Zi-jun Wang, Lian-jie Zhai, Huan Huo, Bo-zhou Wang, Sheng-yong Zhang

**Affiliations:** ^1^State Key Laboratory of Fluorine & Nitrogen Chemicals, Xi'an Modern Chemistry Research Institute, Xi'an, China; ^2^Department of Chemistry, Technische Universität München, Garching bei München, Germany; ^3^Department of Medicinal Chemistry, Fourth Military Medical University, Xi'an, China

**Keywords:** energetic materials, synthesis, 1,2,4-oxadiazole, detonation performances, crystal structure

## Abstract

Design and synthesis of new compounds with both high detonation performances and good safety properties have always been a formidable task in the field of energetic materials. By introducing -ONO_2_ and -NHNO_2_ moieties into 1,2,4-oxadiazole- and 1,2,5-oxadiazole-based backbones, a new family of energetic materials, including ammonium 3-nitramino-4-(5-hydroxymethyl-1,2,4-oxadiazol-3-yl)-furazan (4), 3,3′-bis[5-nitroxymethyl-1,2,4-oxadiazol-3-yl]-4,4′-azofuroxan (6), [3-(4-nitroamino-1,2,5-oxadiazol-3-yl)-1,2,4-oxadiazol-5-yl]-methylene nitrate (8), and its energetic ionic salts (10–12), were synthesized and fully characterized. The energetic and physical properties of the materials were investigated through theoretical calculations and experimental determination. The results show that the oxadiazole-based compounds exhibit high enthalpy of formations, good detonation performances, and extraordinary insensitivities. In particular, the hydrazinium salt (11) shows the best energetic properties (11: *d* = 1.821 g cm^−3^; *P* = 35.1 GPa, *v*_D_ = 8,822 m s^−1^, IS = 40 J, FS > 360N). The ESP and Hirshfeld surface analysis indicated that a large number of hydrogen bonds as well as π-π stacking interactions within molecules might be the key reason for their low sensitivities and high energy-density levels.

## Introduction

**Scheme 1:**
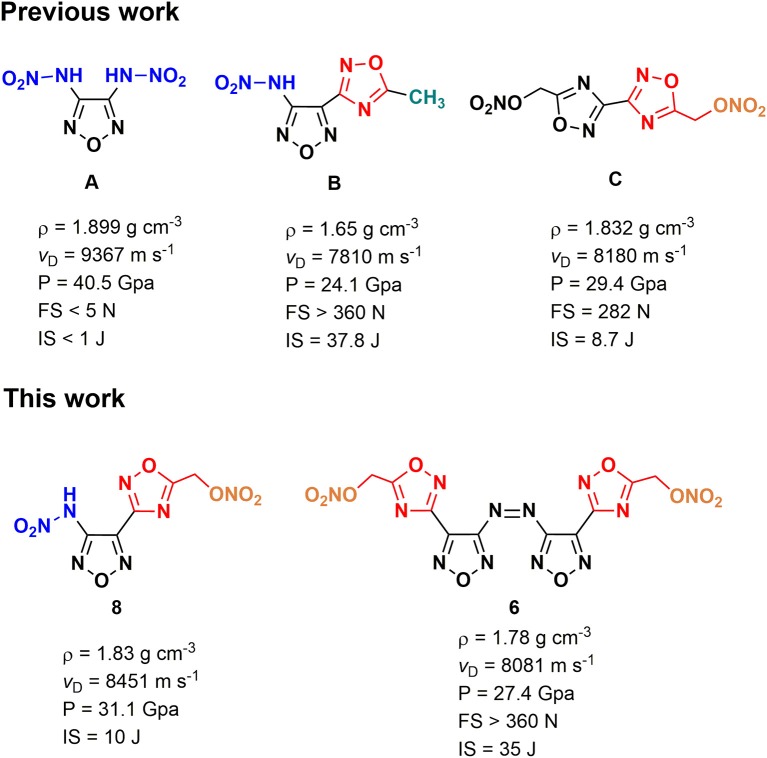
**(A–C)** Electrostatic potential of compounds **6** and **8** [B3LYP/6-31+G** 0.001 electron/b3 isosurface, energy values −0.03 to +0.03 H].

**Figure 1:**
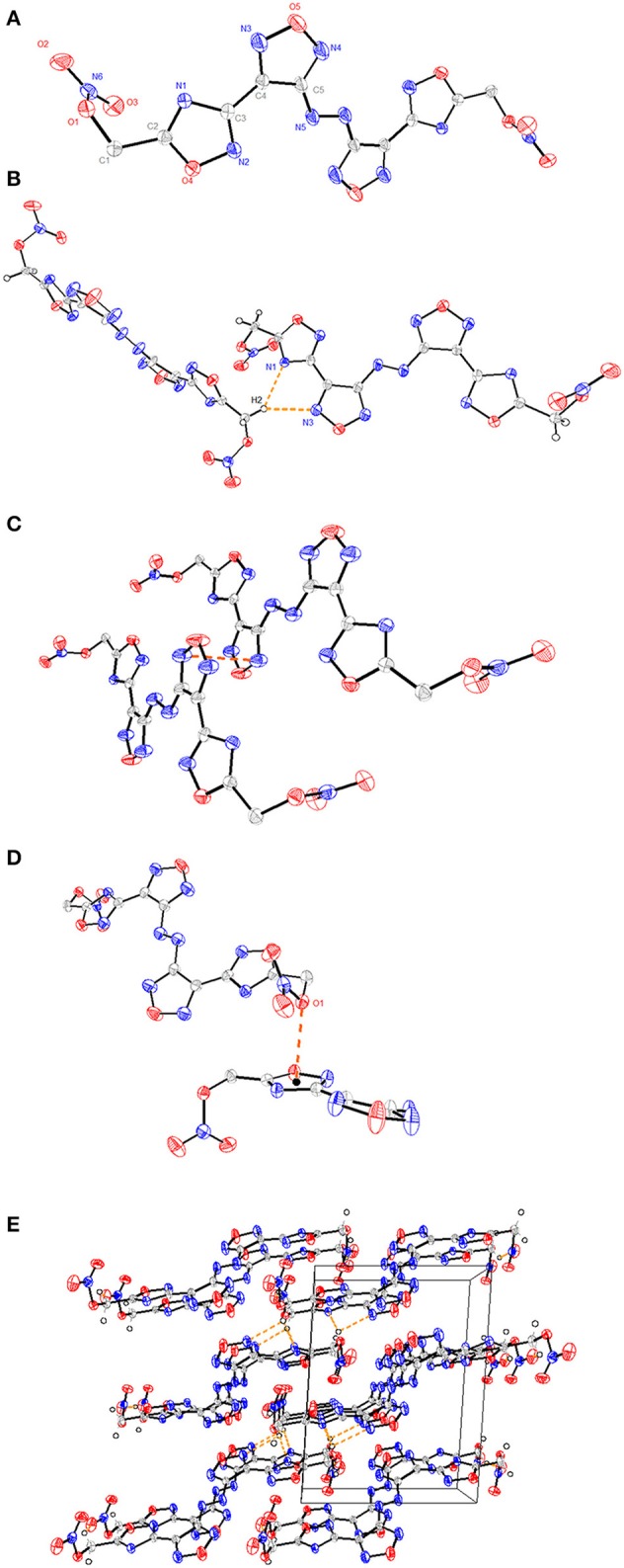
**(A)** A representation of the structure of compound **6** with ellipsoids presented with 30% probability level. **(B)** The packing of compound **6** viewed down the *a*-axis with the weak CH···N hydrogen bonds. **(C)** The π-π stacking interactions between N-heterocyclic rings. **(D)** The lone pair–π interactions between O1 and C3-N2-C2-O4-N3 ring. **(E)** The 3D supramolecular network of compound **6**. The orange dashed lines represent hydrogen bonds.

Nitrogen-rich azoles are widely applied heterocyclic frameworks in the design of energetic materials, among which 1,2,5-oxadiazole (furazan) is lucubrated due to its high heat of formation and good oxygen balance (Zheng et al., 2010; Wang et al., 2011; Fischer et al., 2014; Tsyshevsky et al., 2015; Liu et al., 2018). By incorporating energy-rich functional groups (-NO_2_, -NHNO_2_, -ONO_2_, etc.) into furazan backbones, a large number of energetic materials with excellent detonation properties were successfully developed (Tang et al., 2016; Liu et al., 2018; Zhai et al., 2019; Zhang et al., 2019). Meanwhile, it is noteworthy that an inevitable and inherent contradiction existed between energy and safety of energetic materials, which means higher energy usually goes with lower safety and vice versa. During the past decades, the design and synthesis of new structures with optimal balance between high detonation performances and good safety properties have become a formidable task in the research field of energetic materials (Fischer et al., 2014; Wei et al., 2015). 3,4-Bis(nitramino)-furazan (Scheme 1A), a typical example that has a high density of 1.899 g cm^−3^, is impossible to be applied due to its high sensitivities (IS < 1 J and FS < 5 J) (Figure 1A) (Tang et al., 2015). Although the replacement of a nitramino group with a 5-methyl-1,2,4-oxadiazole (Scheme 1B) moiety can evidently improve the insensitivity (IS = 37.8 J and FS > 360 N), the energetic level of 3-nitramino-4-(5-methyl-1,2,4-oxadiazol-3-yl)furazan methyl(-CH_3_) becomes much lower (ρ = 1.65 cm^−1^ and *v*_D_ = 7,810 m s^−1^) due to the existence of non-energetic methyl group (Yu et al., 2017) (Figure 1B).

Nitrate ester group (-ONO_2_) is a classical energetic group that can improve the oxygen balance and the density of the target compound (Gaur et al., 2017; Stark et al., 2019). Some nitrate ester-based compounds, such as pentaerythritol tetranitrate (PETN) (Klapötke et al., 2007; Li et al., 2012; Srinivas and Ghule, 2016) and nitroglycerin (NG) (Chavez et al., 2008; Davis, 2016), are the most widely used energetic ingredients in civilian and military fields (Politzer and Murray, 2003; Agrawal and Hodgson, 2006; Agrawal, 2010). However, the O–N bond in the -ONO_2_ group is weak as a result of an unbalanced electrostatic potential surface, leading to the high sensitivity and low thermal stability of aliphatic nitrate esters (Qiu et al., 2009). Interestingly, some N-heterocyclic-based energetic nitrate esters exhibit similar detonation performance but higher stability than PETN (Zhang et al., 2016a). For example, bis(1,2,4-oxadiazole)bis(methylene) dinitrate (Scheme 1C), a structure containing two 1,2,4-oxadiazole rings and two nitrate ester groups, shows good energy-density level (ρ = 1.832 g cm^−3^, *v*_D_ = 8180 m s^−1^) (Figure 1C) as well as acceptable decomposition temperature (200.3°C) (Johnson et al., 2018) and sensitivities (IS = 8.7 J and FS = 282 N). Computational chemistry also has proved that the existence of the different oxadiazole rings in an energetic molecule is an effective strategy to reduce sensitivity and achieve good detonation performance (Dippold et al., 2013), which may be due to the conjugated effect. During our research on energetic nitrate esters, we presumed that when nitrate ester groups connected with a backbone based on the combination of 1,2,4-oxadiazole and 1,2,5-oxadiazole, energetic structures with high energy and good safety might be achieved. Based on that, a series of new energetic nitrate esters were designed. Herein, we reported the first synthesis of a family of energetic materials (**4**, **6**, **8**, **10–12**) via cyclization, oxidation, hydrolysis, and nitration procedures. The structures of target compounds and intermediates were characterized by ^1^H NMR, ^13^C NMR, FT-IR, and elementary analysis. The structures of compound **4** • H_2_O and compound **6** were further confirmed by single crystal X-ray diffraction. The energetic properties, thermal stabilities, and sensitivities of the energetic materials were also studied by the theoretical calculations or experimental methods. Furthermore, ESP and Hirshfeld surface analysis were carried out to explore possible reasons for the low sensitivity and high energy.

## Materials and Methods

All chemicals and solvents were obtained from Aladdin Bio-Chem Technology CO. Ltd (Shanghai, China) and used without further purification. 1,2,4-Oxadiazole-3-carboxyamidoxime (1) was supplied by Xi'an Modern chemistry Research Institute. ^13^C and ^1^H NMR spectra were recorded at 298 K on an AV 500 NMR spectrometer (Bruker, Switzerland). Infrared spectra were measured by an EQUINOX 55 Fourier transform infrared spectrometer (Bruker, Germany). Elemental analyses were obtained on the vario EL cube elemental analyzer (Elmentar, Germany). The thermal analysis experiments were recorded on a model TG-DSC STA 499 F3 instrument (NETZSCH, Germany) with dynamic nitrogen atmosphere at a heating rate of 10°C min^−1^. Single crystal X-ray experiment was carried out on a Bruker Apex II CCD diffractometer equipped with graphite monochromatized Mo Kα radiation (λ = 0.71073 Å) using ω and φ scan mode. Structures were solved by the direct method using SHELXTL and refined by means of full-matrix least-squares procedures on F2 with the programs SHELXL-97. Detonation velocity and detonation pressure data were calculated by program package EXPLO5 (version 6.02). The sensitivity data were determined according to BAM standards by BAM drophammer and BAM friction tester (NATO, 1999, 2002).

CCDC number of compound **4** • H_2_O and compound **6** is 1958390 and 1958391, respectively.

### The Calculation Method of the Heats of Formation

All quantum chemical calculations were carried out using the program package GAUSSIAN 09 (Frisch et al., 2009). The geometry optimizations of the molecules and frequency analyses were accomplished by using the B3LYP with the 6-311+G** basis set (Calais, 1993). The gas-state enthalpies and energies of formation of molecule, cations, and anions were calculated using the quantum chemical CBS-4M calculations (H_CBS−4M_) method (Ochterski et al., 1996; Montgomery et al., 2000) in order to obtain accurate values.

The heats of formation of ionic salts can be simplified by using Equation (1) based on Born–Haber energy cycles (Jenkins et al., 2002). The Δ*H*_L_ value is the lattice energy of the ionic salt that can be predicted by Equation (2) (Jenkins et al., 2002), in which *U*_POT_ is the lattice potential energy and *n*_M_ and *n*_X_ depend on the nature of the ions M_*p*_^+^ and X_*q*_^−^, respectively, and are equal to three for monoatomic ions, five for linear polyatomic ions, and six for nonlinear polyatomic ions. The value of *U*_POT_ can be obtained through Equation (3) (Jenkins et al., 2002), in which ρ_m_ is the density (in g cm^−3^), *M*_m_ is the chemical formula mass of the ionic material (in g), and the coefficients γ (in kJ mol^−1^ cm) and δ (in kJ mol^−1^) are assigned literature values.

(1) $$\begin{array}{l} \Delta H_{f}^{0}\left(\text{ionic salt},\;298\text{ K}\right)=\Delta H_{f}^{0}\left(\text{cation},\;298\text{ K}\right)\\ \;+\Delta H_{f}^{0}\left(\text{ation},\;298\text{ K}\right)-\Delta H_{L} \end{array}$$

(2) $$\begin{array}{l} \Delta H_{L}=U_{\text{POT}}+\left[\rho \left(\frac{n_{M}}{2}-2\right)+q\left(\frac{n_{X}}{2}-2\right)\right]RT \end{array}$$

(3) $$\begin{array}{l} U_{\text{POT}}={\gamma \left(\rho_{m}/M_{m}\right)}^{1/3}+\delta \end{array}$$

### Synthesis

#### [3-(4-Amino-1,2,5-oxadiazol-3-yl)-1,2,4-oxadiazol-5-yl]-methylene Acetate (2)

The 2-chloro-2-oxoethyl acetate (3.43 g, 23.97 mmol) in the 20-ml mixed solvent of anisole and toluene (v/v = 1:5) was added dropwise to a stirred mixture of 1,2,4-oxadiazole-3-carboxyamidoxime (**1**) (3.60 g, 26.37 mmol) and pyridine (3.81 g, 31.45 mmol) at room temperature. After complete addition, the resulting solution was heated to 135°C for 4 h. The filtrate was collected by Büchner funnel to yield the desired compound **2** as white solid. Yield: 4.8 g, 89 wt%. ^1^H NMR (500 MHz, DMSO-*d*_6_) δ6.50 (s, 2H), 5.54 (s, 2H), 2.18 (s, 3H). ^13^C NMR (126 MHz, DMSO-*d*_6_) δ 176.68, 170.27, 159.49, 155.89, 137.29, 56.87, 20.63. IR (KBr), ṽ, 3,475, 3,364, 2,953, 1,756, 1,630, 1,590, 1,549, 1,438, 1,388, 1,355, 1,251, 1,222, 1,149, 1,071, 1,016, 972, 928, 864. Anal. Calcd for C_7_H_7_N_5_O_4_ (%), C, 37.34; H, 3.13; N, 31.10. Found (%), C, 37.25; H, 3.25; N, 31.09 (Figures S1, S2, and S20).

#### 3,3'-Bis(5-hydroxymethyl−1,2,4-oxadiazole-3-yl)-4,4'-azofuroxan (5)

The saturated aqueous solution of KMnO_4_ (1.41 g, 8.92 mmol) was added dropwise to a solution of 1 g of compound **2** (2.00 g, 8.88 mmol) in 13 ml of conc. hydrochloric acid at ambient temperature. The mixture was warmed to 50°C and stirred for another 4 h. The precipitate was filtered off and dried by air flow as an orange powder. Yield: 2.51 g, 78 wt%. ^1^H NMR (500 MHz, DMSO-*d*_6_) δ 6.12 (s, 2H), 4.86 (s, 4H). ^13^C NMR (126 MHz, DMSO-*d*_6_) δ 181.40, 162.26, 157.96, 141.78, 55.55. IR (KBr), ṽ, 3,384, 2,925, 1,752, 1,581, 1,466, 1,434, 1,397, 1,349, 1,241, 1,147, 1,081, 977, 916, 875. Anal. Calcd for C_10_H_6_N_10_O_6_ (%), C, 33.16; H, 1.67; N, 38.67. Found (%), C, 33.50; H, 2.085; N, 38.22 (Figures S6, S7, and S22).

#### 3-Amino-4-(5-hydroxymethyl-1,2,4-oxadiazol-3-yl)-furazan (7)

Compound **2** (2.00 g, 8.88 mmol) was added in portions to a mixture of KHCO_3_ (0.26 g, 2.66 mmol) and methanol (150 ml) at room temperature. The reaction mixture was heated to reflux, stirred for 2 h, and then cooled to room temperature. The reaction mixture was concentrated in vacuo to give a crude solid. The pure production was obtained and purified with 20 ml of H_2_O and collected by Büchner filtration. Light yellow solid. Yield: 1.55 g, 95 wt%. ^1^H NMR (500 MHz, DMSO-*d*_6_) δ 6.84 (s, 2H), 5.71 (s, 1H), 5.48 (s, 2H). ^13^C NMR (126 MHz, DMSO-*d*_6_) δ 181.40, 160.42, 156.47, 137.77, 56.63. IR (KBr), ṽ, 3,467, 3,385, 3,319, 2,929, 1,642, 1,604, 1,583, 1,557, 1,434, 1,400, 1,376, 1,242, 1,154, 1,089, 374, 905, 875, 741. Anal. Calcd for C_10_H_6_N_10_O_6_ (%), C, 33.16; H, 1.67; N, 38.67. Found (%), C, 33.50; H, 2.28; N, 38.22 (Figures S10, S11, and S23).

### General Procedure for Compounds 3, 6, and 8

Fifteen milliliters of 100% HNO_3_ was cooled to 0°C. The reactant (compound **2**, compound **5**, or compound **7**, 1 g) was carefully added to the cooled nitric acid (15 ml) while maintaining the temperature at ≤ 5°C. After complete addition, the solution was stirring for 4 h at 5°C. The mixture was poured into ice-cold water followed by extraction with ethyl acetate three times. The organic phases that were washed by water until close to neutralization were evaporated under reduced pressure to obtain the crude product.

#### 3-Nitramino-4-(5-hydroxymethyl-1,2,4-oxadiazol-3-yl)-furazan (3)

Light yellow liquid. Yield: 98 wt%. ^1^H NMR (500 MHz, Acetone-*d*_6_) δ 4.95 (s, 2H). ^13^C NMR (126 MHz, Acetone-*d*_6_) δ 182.10, 164.30, 124.27, 109.40, 57.32. Anal. Calcd for C_7_H_7_N_5_O_4_ (%), C, 26.33; H, 1.77; N, 36.84. Found (%), C, 26.01; H, 2.25; N, 36.09 (Figures S3 and S4).

#### 3,3'-Bis[5-nitroxymethyl-1,2,4-oxadiazol-3-yl]-4,4'-azofuroxan (6)

Orange solid. Yield: 85 wt%. ^1^H NMR (500 MHz, Acetone-*d*_6_) δ 6.13 (s, 2H) ^13^C NMR (126 MHz, Acetone-*d*_6_) δ 175.88 (s), 163.14 (s), 159.35 (s), 141.16 (s), 64.78(s). IR (KBr), ṽ, 3,441, 3,003, 2,959, 1,657, 1,597, 1,464, 1,418, 1,345, 1,294, 1,223, 1,145, 1,053, 986, 913, 849. Anal. Calcd for C_10_H_4_N_12_O_10_ (%), C, 26.56 H, 0.89; N, 37.17. Found (%), C, 26.54; H, 1.38; N, 37.25 (Figures S8 and S9).

#### [3-(4-Nitroamino-1,2,5-oxadiazol-3-yl)-1,2,4-oxadiazol-5-yl]-methylene nitrate (8)

Light yellow liquid. Yield: 95 wt%. ^1^H NMR (500 MHz, Acetone-*d*_6_) δ 6.17 (s, 2H). ^13^C NMR (126 MHz, Acetone-*d*_6_) δ 175.74, 159.15, 149.19, 143.16, 64.47. IR (KBr), ṽ, 3,568, 3,134, 2,964, 1,618, 1,551, 1,476, 1,431, 1,314, 1,217, 1,150, 1,069, 971, 918, 838, 751. Anal. Calcd for C_5_H_3_N_7_O_7_ (%), C, 21.99; H, 1.11; N, 35.90; O, 41.00. Found (%), C, 22.05; H, 1.35; N, 36.12 (Figures S12, S13, and S24).

#### Ammonium 3-nitramino-4-(5-hydroxymethyl-1,2,4-oxadiazol-3-yl)-furazan (4)

Compound **3** (1 g) was dissolved in MeOH (2 ml), and aqueous ammonia (28 wt% in water) was added dropwise until pH = 8. The mixture was stirred for 2 h at room temperature, the precipitate was filtered off as yellow solid. Yield: 0.96 g, 90 wt%. ^1^H NMR (500 MHz, D_2_O) δ 4.96 (s), 4.70 (s). ^13^C NMR (126 MHz, D_2_O) δ 180.19, 158.56, 155.11, 141.26, 55.40. IR (KBr), ṽ, 3,195, 3,085, 1,658, 1,594, 1,504, 1,419, 1,272, 1,151, 1,048, 1,010, 967, 823. Anal. Calcd for C_5_H_7_N_7_O_5_ (%), C, 24.50; H, 2.88; N, 39.99; O, 32.63. Found (%), C, 24.54; H, 2.78; N, 40.05 (Figures S5 and S21).

#### Silver [3-(4-nitroamino-1,2,5-oxadiazol-3-yl)-1,2,4-oxadiazol-5-yl]-methylene Nitrate (9)

Compound **8** (2 mmol) was dissolved in the mixed solvent of water and methanol (5 ml, 1:1). Silver nitrate (2.1 mmol) was dissolved in water (5 ml) and carefully added to a solution of compound **8**. After stirring for 2 h at room temperature, the product was filtered off as a yellow solid. Yield: 1.36 g, 98 wt%. IR (KBr), ṽ, 3,456, 3,004, 2,964, 1,658, 1,587, 1,446, 1,274, 1,156, 1,076, 817. Anal. Calcd for C_5_H_7_N_7_O_5_ (%), C, 24.50; H, 2.88; N, 39.99; O, 32.63. Found (%), C, 24.54; H, 2.78; N, 40.05.

### General Procedure for the Preparation of the Salts (10–12)

The reactant (ammonium chloride, hydroxylamine hydrochloride, or biguanide hydrochloride, 3 mmol) was dissolved in water (5 ml) and compound **9** (2 mmol) dissolved in methanol (1 ml) was added. After stirring for 2 h at room temperature, the precipitation was filtered. The solution was evaporated to dryness to obtain the solid.

#### Ammonium [3-(4-nitroamino-1,2,5-oxadiazol-3-yl)-1,2,4-oxadiazol-5-yl]-methylene Nitrate (10)

Light yellow solid. Yield: 75 wt%. ^1^H NMR (500 MHz, CD_3_OD) δ 7.42 (s), 6.09 (s). ^13^C NMR (126 MHz, CD_3_OD) δ 175.29 (s), 158.82 (s), 141.57 (s), 133.61(s), 63.67 (s). IR (KBr), ṽ, 3,145, 3,001, 1,646, 1,535, 1,505, 1,401, 1,288, 1,153, 967, 918, 821. Anal. Calcd for C_5_H_6_N_8_O_7_ (%), C, 20.70; H, 2.08; N, 38.62; O, 38.60. Found (%), C, 20.12; H, 3.35; N, 39.12 (Figures S14, S15, and S25).

#### Hydroxylamine [3-(4-nitroamino-1,2,5-oxadiazol-3-yl)-1,2,4-oxadiazol-5-yl]-methylene Nitrate (11)

White solid. Yield: 85%. ^1^H NMR (500 MHz, CD_3_OD) δ 8.29 (s), 5.99(s). ^13^C NMR (126 MHz, CD_3_OD) δ 175.63 (s), 160.82 (s), 142.87 (s), 135.91(s), 64.70 (s). IR (KBr), ṽ, 3,442, 3,163, 3,080, 2,968, 2,775, 1,664, 1,599, 1,530, 1,506, 1,451, 1,403, 1,280, 1,158, 1,059, 1,011, 968, 834. Anal. Calcd For C_5_H_3_N_7_O_7_ (%), C, 19.62; H, 1.98; N, 36.60; O, 41.81. Found (%), C, 20.05; H, 2.35; N, 37.54 (Figures S16, S17, and S26).

#### Biguanide [3-(4-nitroamino-1,2,5-oxadiazol-3-yl)-1,2,4-oxadiazol-5-yl]-methylene nitrate (12)

Light yellow solid. Yield: 80%. ^1^H NMR (500 MHz, CD_3_OD) δ 7.13 (s), 5.90 (s). ^13^C NMR (126 MHz, CD_3_OD) δ 178.95 (s), 161.06 (s), 160.66 (s), 157.96(s), 143.13 (s), 64.23 (s). IR (KBr), ṽ, 3,355, 3,223, 2,196, 2,154, 1,703, 1,638, 1,545, 1,395, 1,305, 1,154, 1,074, 1,012, 970, 917, 824. Anal. Calcd for C_7_H_10_N_12_O_7_ (%), C, 22.47; H, 2.69; N, 44.91; O, 29.93. Found (%), C, 22.05; H, 3.14; N, 43.12 (Figures S18, S19, and S27).

## Results and Discussion

### Synthesis

**Scheme 2:**
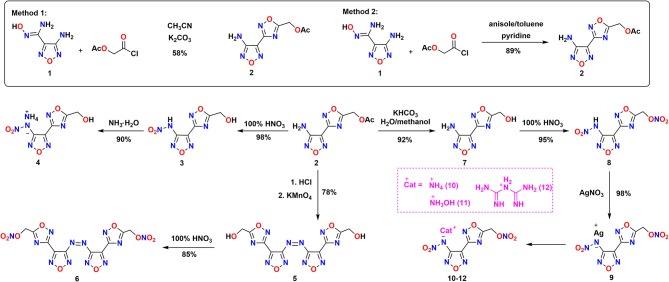
Synthetic pathway of energetic materials (**4, 6, 8, 10–12**) via cyclization, oxidation, hydrolysis, and nitration sequence.

The synthetic pathway to energetic compounds (**4**, **6**, **8**, **10–12**) is shown in Scheme 2. The construction of N-heterocyclic skeleton is the core of energetic materials (Xue et al., 2019). Compound **2** was firstly prepared by 1,2,4-oxadiazole-3-carboxyamidoxime (**1**) and 2-chloro-2-oxoethyl acetate in the K_2_CO_3_/CH_3_CN system to perform cyclization reaction with the procedure analogous to the literature ^12^. The yield of the cyclizing product was only 58% and was purified by chromatographic column. In order to optimize the cyclization reaction, attempts were carried out at high temperatures in anisole/toluene under the catalysis of pyridine with the yield of 89%. By this “one-pot” way, compound **2** could be used in the next step without purification.

Attempted nitrolysis of the acetate groups with two kinds of nitrification conditions, 100% HNO_3_ and 100% HNO_3_/Ac_2_O, afforded no nitrate ester product but compound **3**. In contrast, compound **8** was easily obtained by nitrating the hydrolysis product of **7** with 100% HNO_3_ in 98% yield; it means that an additional hydrolytic step is necessary. When we treated compound **7** with H_2_SO_4_/HNO_3_, a number of new spots were observed by thin-layer chromatography. This may be because the decomposition of the 1,2,4-oxadizazole aromaticity ring was decomposed in the mixed acid system (Zhang et al., 2016b). Treatment of **8** with excess silver nitrate gave **9** as light yellow solid. Subsequently, three kinds of salts **10–12** were obtained by treatment of **9** with ammonium chloride, hydroxylamine hydrochloride, or biguanide hydrochloride.

Most studies show that heterocyclic azoles connected by azo bridges is a good approach to maintain their planar structure and aromaticity (KlapöTke and Piercey, 2011; Tang et al., 2012). When compound **2** was added to the acidic potassium permanganate, azo compound **5** was produced in 78% yield. Similarly, compound **5** was easily transformed to the nitrate compound **6** with 100% HNO_3_.

### Crystal Structure and Weak Interaction Analysis

Crystals of compound 4 • H_2_O and compound 6 were obtained by slow evaporation of methanol/H_2_O at room temperature, and their crystal structures were determined by X-ray diffraction studies (Tables S1–S5). Compound 6 crystallizes in the monoclinic space group, P2_1_/c, with four molecules in each lattice cell and a density of 1.774 g cm^−3^ at 296 (2) K. All four N-heterocyclic skeletons are nearly planar with torsion angles, which can be seen from the torsion angles of N6-C5-C4-C3 (2.16°) and N4-C4-C3-N2 (15.56°) (Figure 1A). The bond lengths of C3-C4 [1.446(7) Å], C1-C2 [1.480(8) Å], and C5-N6 [1.410(7) Å] are shorter than normal carbon–carbon or carbon–nitrogen single bond length (ca. 1.54 Å) markedly (Allen et al., 1987).

Such data illustrate that these aromatic rings form a large conjugated system. However, the two nitrate ester moieties bonded to the methylene linkage are considerably twisted relative to each other with torsion angles of C2-C1-O1-N1 (−77.70°), and C1-O1 bond length [1.435(7) Å] is longer than that of a typical C-O double bond (1.42 Å) (Allen et al., 1987), indicating that the addition of nitrate ester groups may decrease the structure stability. In addition, the intermolecular hydrogen bonds of C1-H1…O2 [3.127(8) Å, 119.0°], C1-H2…N3 [3.480(8) Å, 137.2°], and C1-H2…N1 [3.346(7) Å, 138.2°] were observed in the packing system (Figure 1B). Interestingly, there exsit π-π stacking interactions between N-heterocyclic rings with a separation of ca. 3.53 Å (Figure 1C), and lone pair–π interactions between O1 and C3-N2-C2-O4-N3 ring with a separation of ca. 2.93 Å in the 3D supermolecular structure (Figures 1D,E).

**Figure 2:**
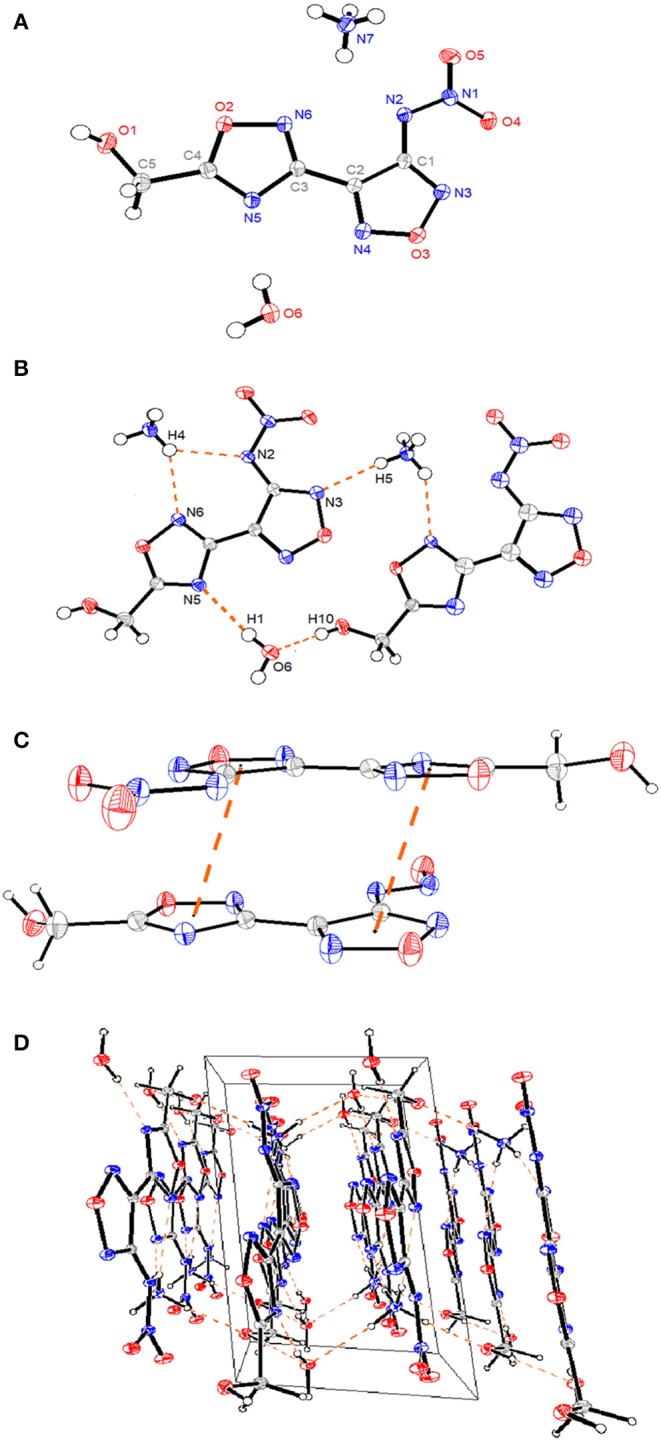
**(A)** A representation of the structure of compound **4** • H_2_O with ellipsoids presented with 30% probability level. **(B)** The packing of compound **4** • H_2_O viewed down. **(C)** The π-π stacking interactions between N-heterocyclic rings. **(D)** The 3D supramolecular network of compound **4** • H_2_O. The orange dashed lines represent hydrogen bonds.

Compound **4** • H_2_O crystallizes in the triclinic space group P-1 with two molecules in the asymmetric unit and a density of 1.66 g cm^−1^ [296 (2) K]. Similar to compound **6**, the whole molecule exhibits a planar structure (Figure 2A), which is reflected in the torsion angles of almost 0° or 180° [N3-C1-N2-N1 (−3.01°), C3-C2-C1-N2 (−0.64°), and C1-N2-N1-O5 (179.04°)] except for the methylene group. The 1,2,4- and 1,2,5-oxadiazole rings are linked by C2-C3 with a C–C bond length of 1.459(2) Å, which is shorter than that of a typical C–C bond (1.54 Å). The crystal structures of compound **4** present a three-dimensional supermolecular structure through intra-/inter-extensive hydrogen-bonding interactions among water molecules, ammonium ions, and the anions in the range of 2.725–3.340 Å (the distances of N-H···O, N-H···N, O-H···O, O-H···N, C-H···O) (Figures 2B,C). Besides, parts of π-π stacking interactions with a separation of ca. 3.23 Å between N-heterocyclic rings are observed (Figure 2D). All these observations are related with its high density, low sensitivity, and excellent thermal stability.

**Figure 3:**
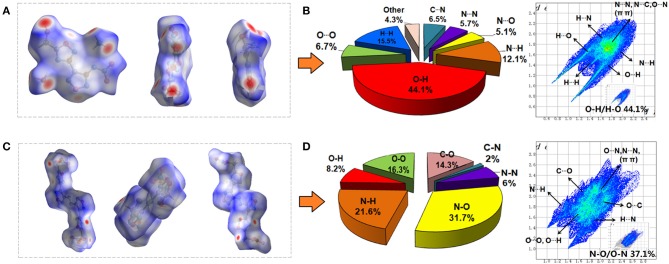
The Hirshfeld surfaces for **(A)** compound **4** • H_2_O and **(C)** compound **6** molecules (white, distance *d* = the van der Waals distance; blue, *d* > the van der Waals distance; red, *d* < van der Waals distance); 2D fingerprint plots in crystal stacking and individual atomic contacts percentage in the bar graphs for compound **4 (B)** and **6 (D)**.

To gain further information about weak inter-/intramolecular interaction in compound 4 • H_2_O and 6, 2D fingerprints and the associated Hirshfeld surfaces of single crystals were fully investigated (Figure 3). As shown in Figures 3A,C, both compound molecules presented as almost planar shapes with some red dots. In typical Hirshfeld surface analysis, the bright red dots on the edges of surfaces denote the high close contact from the 2D spread of the intermolecular HBs, such as N-H/H-N and O-H/H-O interactions (Hu et al., 2019; Wang et al., 2019; Xiong et al., 2019). Compared with compound 6, the Hirshfeld surfaces of compound 4 has more bright red dots, which means that the intermolecular hydrogen bonds of compound 4 is stronger than compound 6. Analysis of 2D fingerprints data shows that the intermolecular hydrogen bonds in the packing system of compounds 4 and 6 were 56.2 and 29.8% of the total weak interactions, respectively (Figures 3B,D). However, for compound 6, non-HBs contact, but the π-π and p–π stacking accounts for 52% of the total weak interactions, which reflects a high percentage of interlayer contact.

### Sensitivities and Thermal Stability

**Figure 4:**
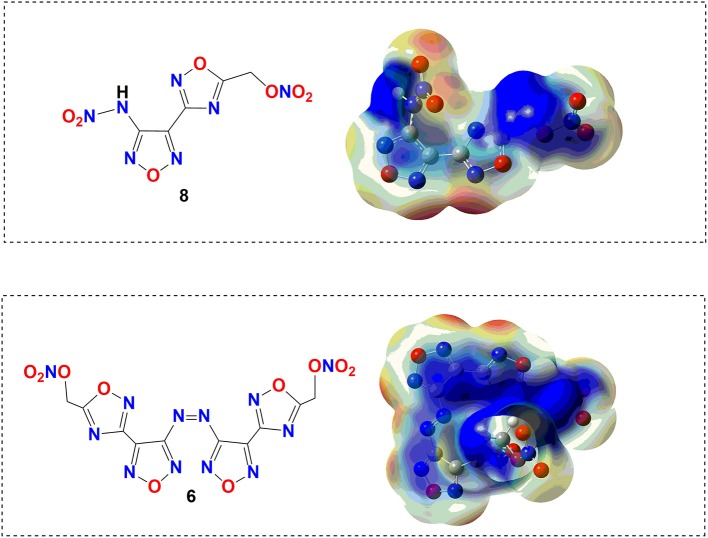
Several relative energetic compounds and their detonation performances.

To obtain further understanding of the safety performance in the level of molecules for target compounds, electrostatic potential (ESP), the effective theoretical calculation method to predict sensitivities of energetic materials, was carried out based on the B3LYP/6-31+G** method by Gaussian 09 software (Frisch et al., 2009) with optimized structure before the actual measurement of the sensitivity. In theory, a higher charge separation and larger and stronger positive potential will cause higher impact sensitivity. It can be clearly seen from Figure 4 that the potential on compound **6** is uniformly dispersed roughly because strong π-π stacking and lone pair–π interactions exist in its structure. Compared with compound **6**, the charges of nitramino (–NH–NO_2_) in compound **8** are obviously more positive, ultimately resulting in an imbalance of charge distribution. Therefore, we can be concluded that compound **6** is more stable than compound **8**.

The ESP results were confirmed by the experimental method. Impact and friction sensitivities were measured using standard BAM techniques; the experimental results are listed in Table 1. The impact sensitivities of compounds **6** and **8** are 35 and 10 J, respectively. However, the mechanical sensitivity of compound **8** could be greatly increased by forming energetic salt with nitrogen-containing cations. Impact sensitivities of compounds **10–12** are in the range of 35 J (**10**) and >40 J (**12**), and the FS values of all the salts are higher than 360 N. The result illustrated, except for **8**, that all energetic compounds are classified as “less sensitive” or “insensitive” according to the UN guidelines^1^.

**Table 1:** Energetic properties of compounds **6**, **8**, and **10**–**12**.

**Compound**	***T*_m_^a^ (°C)**	***T*_d_^b^ (°C)**	**ρ (g cm^**−3**^)**	**Δ*_***f***_*H^e^ (kJ mol^**−1**^)**	****v*_***D***_*^f^ (m s^**−1**^)**	***P*^h^ (GPa)**	***IS*^i^ (J)**	***FS*^j^ (N)**
6	135	200	1.78^c^	650.7	8,081	27.4	35	>360
8	–	60	1.83^d^	237.8	8,451	31.1	10	–
10	–	135	1.79^d^	253.5	8,428	32.0	35	>360
11	–	148	1.82^d^	309.1	8,822	35.2	40	>360
12	–	135	1.75^d^	263.1	7,809	27.2	>40	>360
TNT^23^		295	1.65	−59.4	6,881	19.5	15	240
RDX^23^		204	1.80	92.6	8,795	34.9	7.5	120

a *The melting point (DSC, 10°C min^−1^)*.

b *The decomposition point (DSC, 10°C min^−1^)*.

c *Single crystal density at 296 (2) K*.

d *Measured density at 294 (2) K*.

e *Calculated heat of formation*.

f *Calculated detonation velocities*.

h *Calculated detonation pressure*.

i *Impact sensitivities*.

j *Friction sensitivity*.

Furthermore, compounds were tested for thermal capacity in DSC measurements (10°C min^−1^) using dry nitrogen in the temperature range from 50 to 350°C. As shown in Table 1, most compounds decompose without melting. Among them, azo compound **6** exhibits the best thermal stability with a decomposition temperature at 200°C.

### Detonation Performance

Detonation performance is one of the most important factors in evaluating the performance of energetic compounds, which is closely related to the density and the heats of formation (Δ_*f*_H). The densities of all compounds, measured with a gas pycnometer at 25°C, are in the range of 1.75 cm^−3^ to 1.83 cm^−3^. The densities of compound **8** and its hydroxylammonium salt **11** are more than 1.80 g cm^−3^, which is superior to RDX. The heats of formation (Δ_*f*_H) calculated results are shown in Table 1. Compounds **6** and **10–12** have relatively high positive heats of formation (Δ_*f*_H) ranging from 137.8 and 657.0 kJ mol^−1^, among which compound **10** has the lowest value of 253.5 kJ mol^−1^, twice higher than that of RDX (92.6 kJ mol^−1^), and compound **6** has the highest heat of formation (650.7 kJ mol^−1^), indicating that the azo group can increase the heats of formation of energetic compounds.

Based on the experimental densities and calculated solid-phase heats of formation, calculation of the detonation pressure (P) and velocity (*v*_D_) was performed using the EXPLO5 v6.01 program (Suceska, 2013). The detonation performances of compounds **6**, **8**, and **10–12** are listed in Table 1. The calculated detonation velocities lay between 7,809 and 8,822 m s^−1^, and the calculated detonation pressures are between 27.2 and 35.2 GPa (Table 1). Compound **11** exhibited the optimal performance parameter (*P* = 35.2 GPa, *D* = 8,822 m s^−1^) among these compounds, slightly better than those of RDX (*P* = 34.9 GPa, *D* = 8,795 m s^−1^). The calculated properties coupled with the rather high thermal and hydrolytic stabilities suggest these high nitrogen materials as attractive candidates for insensitive energetic applications.

## Conclusions

In summary, a strategy to design potential insensitive energetic materials with high denotation performance was presented in this study. By the combination of C-ONO_2_ high energetic moiety, 1,2,4-oxadiazole, and furazan conjugate insensitive backbones, a family of energetic materials (**4, 6, 8, 10–12**) were designed, synthesized, and fully characterized. As expect, the prepared energetic materials show promising properties such as high density (ρ = 1.75–1.83 g cm^−3^), excellent detonation performance (*v*_D_ = 7,809–8,822 m s^−1^, *P* = 27.2–35.2 GPa), and low sensitivities (IS > 35 J, FS > 360 N except compound **8**). Among them, **11** has a good measured density of 1.821 g cm^−3^, high detonation properties (*v*_D_ = 8,822 m s^−1^, *P* = 35.2 GPa), and low sensitivities (IS = 35 J and FS > 360 N). Further ESP and Hirshfeld surface analysis indicates that the presence of a large number of hydrogen bonds and π-π stacking in this series of energetic compounds may be an important reason for their low sensitivity and high energy. The result confirmed that the strategy of introducing a nitrogen-rich heterocyclic ring with moderate enthalpy of formation into a nitrogen-rich heterocycle system with high enthalpy of formation and constructing a conjugated system is a powerful path to develop new high-performing insensitive materials.

## Data Availability Statement

The datasets generated for this study can be found in the Cambridge Crystallographic Data Centre (https://www.ccdc.cam.ac.uk/structures/) under the identifiers 1958390 and 1958391.

## Author Contributions

BW and SZ conceived the experiments. QX and FB conducted the experiments. QX wrote the first draft of the manuscript. ZW and HH ran calculations. JZ and LZ revised and edited the manuscript. All authors contributed to manuscript revision, read, and approved the submitted version.

### Conflict of Interest

The authors declare that the research was conducted in the absence of any commercial or financial relationships that could be construed as a potential conflict of interest.

## Footnotes

^1^UN Guidelines. Impact: insensitive 40 J, less sensitive 35 J, sensitive 44 J, very sensitive 43 J. Friction: insensitive >360 N, less sensitive 360 N, sensitive <360 N, very sensitive >380 N, extremely sensitive >310 N. According to the UN Recommendations on the Transport of Dangerous Goods, (+) indicates “not safe for transport.”
