# Supplementary material for: A Family of Energetic Materials Based on 1,2,4-Oxadiazole and 1,2,5-Oxadiazole Backbones With Low Insensitivity and Good Detonation Performance
Source: Front Chem. 2020 Feb 20;7:942. doi: 10.3389/fchem.2019.00942 (PMC7044674; doi:10.3389/fchem.2019.00942)
Supplement: Supplementary file 1 [file Data_Sheet_1.pdf]

frontiers

Supplementary Material

Table S1. Crystal data and structure refinement parameters of compound **4**•H_2O and compound **6**.

Compd	compound **4**•H_2O	compound **6**
Identification code	1	2
Empirical Formula	$C_5H_9N_7O_6$	$C_5H_2N_6O_5$
Molecular mass	263.19	226.13
Crysta system	triclinic	Monoclinic
Space group	P-1	$P2_1/c$
a/Å	6.6361(7)	10.111(12)
b/Å	7.6567(7)	6.167(7)
c/Å	10.5686(11)	13.608(17)
α/°	86.461(2)	90.00
β/°	82.057(2)	93.56(2)
γ/°	83.5520(10)	90.00
Volume/$Å^3$	527.90(9)	846.8(18)
Z	2	4
Temperature(K)	296(2)	296(2)
ρ_{calc} g / cm^3	1.656	1.774
μ / mm^{-1}	0.146	0.160
F(000)	264.0	456.0
Crystal size/mm^3	0.10 × 0.10 × 0.10	0.28 × 0.20 × 0.05
Radiation	MoKα (λ = 0.71073)	MoKα (λ = 0.71073)
Goodness-of-fit on F^2	1.022	1.063
Final R indexes [I>=2σ (I)]	R_1 = 0.0327, wR_2 = 0.0810	R_1 = 0.0774, wR_2 = 0.1863
Final R indexes [all data]	R_1 = 0.0389, wR_2 = 0.0845	R_1 = 0.1027, wR_2 = 0.2040
Data/restraints/parameters	1852/0/188	1410/0/154
Index ranges	$-7 \le h \le 7$, $-9 \le k \le 8$, $-12 \le l \le 10$	$-11 \le h \le 5$, $-6 \le k \le 7$, $-16 \le l \le 16$

Table S2. Bond lengths for compound **6**.

Atom	Atom	Length/Å	Atom	Atom	Length/Å
C5	N4	1.301(7)	C2	C1	1.482(7)
C5	N5	1.408(7)	C1	O1	1.435(6)
C5	C4	1.414(6)	N5	N5[1]	1.209(8)
C4	N3	1.297(7)	N4	O5	1.370(6)
C4	C3	1.446(7)	N3	O5	1.376(6)
C3	N2	1.290(6)	N2	O4	1.396(5)
C3	N1	1.367(6)	N6	O3	1.189(6)
C2	N1	1.292(6)	N6	O2	1.202(6)
C2	O4	1.330(6)	N6	O1	1.391(5)

Table S3. Bond angles for compound **6**.

Atom	Atom	Atom	Angle/°	Atom	Atom	Atom	Angle/°
N4	C5	N5	124.5(4)	O1	C1	C2	110.3(4)
N4	C5	C4	110.1(5)	N5[1]	N5	C5	114.3(6)
N5	C5	C4	125.3(4)	C5	N4	O5	104.5(4)
N3	C4	C5	108.4(4)	C4	N3	O5	105.5(4)
N3	C4	C3	121.2(4)	C2	N1	C3	101.4(4)
C5	C4	C3	130.4(5)	C3	N2	O4	102.7(4)
N2	C3	N1	115.7(4)	O3	N6	O2	129.5(5)
N2	C3	C4	121.4(4)	O3	N6	O1	118.9(5)
N1	C3	C4	122.9(5)	O2	N6	O1	111.6(5)
N1	C2	O4	113.7(4)	N4	O5	N3	111.5(4)
N1	C2	C1	128.9(5)	C2	O4	N2	106.5(4)
O4	C2	C1	117.4(4)	N6	O1	C1	112.5(4)

Table S4. Bond lengths for compound **4**•H_2O.

Atom	Atom	Length/Å	Atom	Atom	Length/Å
C1	N3	1.314(2)	C4	C5	1.490(2)
C1	N2	1.368(2)	C5	O1	1.398(2)
C1	C2	1.436(2)	N1	O5	1.2354(19)
C2	N4	1.299(2)	N1	O4	1.2534(18)
C2	C3	1.459(2)	N1	N2	1.3286(19)
C3	N6	1.298(2)	N3	O3	1.3999(19)
C3	N5	1.372(2)	N4	O3	1.3563(18)
C4	N5	1.293(2)	N6	O2	1.4082(17)
C4	O2	1.3334(19)			

Table S5. Bond angles for compound **4**•H_2O.

Atom	Atom	Atom	Angle/˚	Atom	Atom	Atom	Angle/˚
N3	C1	N2	131.82(15)	O1	C5	C4	111.04(15)
N3	C1	C2	107.76(14)	O5	N1	O4	120.63(14)
N2	C1	C2	120.41(14)	O5	N1	N2	116.75(14)
N4	C2	C1	109.86(14)	O4	N1	N2	122.61(14)
N4	C2	C3	118.40(14)	N1	N2	C1	116.51(13)
C1	C2	C3	131.72(14)	C1	N3	O3	105.11(13)
N6	C3	N5	115.39(14)	C2	N4	O3	105.76(13)
N6	C3	C2	123.06(14)	C4	N5	C3	101.98(13)
N5	C3	C2	121.56(14)	C3	N6	O2	102.52(12)
N5	C4	O2	113.47(14)	C4	O2	N6	106.63(12)
N5	C4	C5	127.85(15)	N4	O3	N3	111.50(12)
O2	C4	C5	118.66(15)				

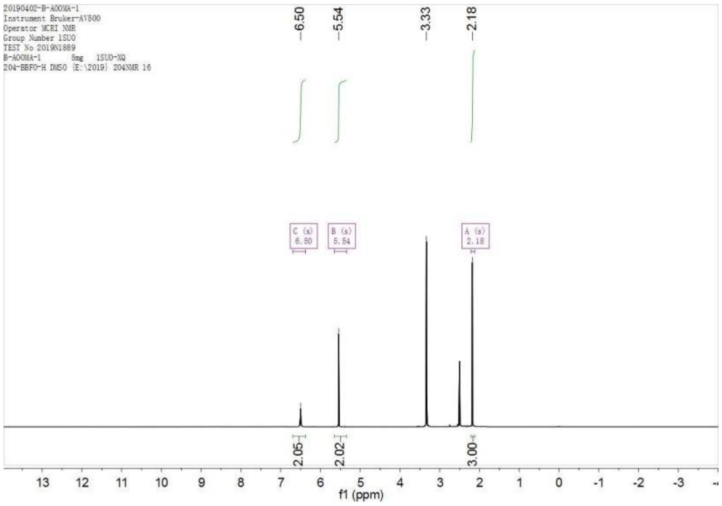

Fig. S1. ^{1}H NMR (500 MHz, DMSO-d_6) data of compound **2.**

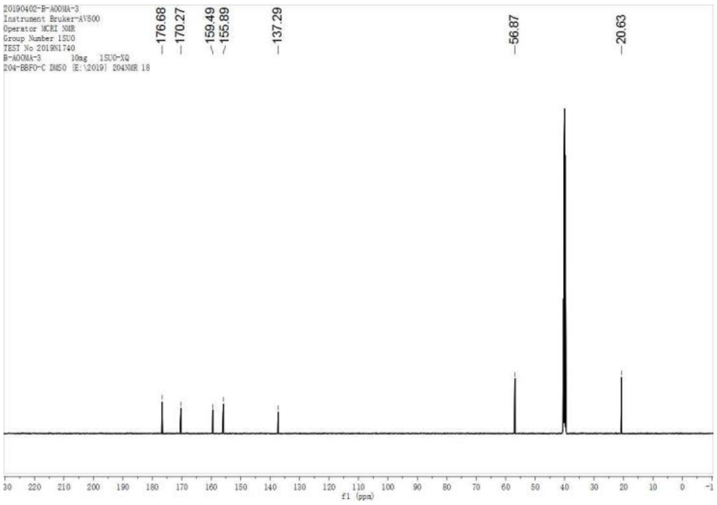

Fig. S2. ^{13}C NMR (126 MHz, DMSO-d_6) data of compound **2**.

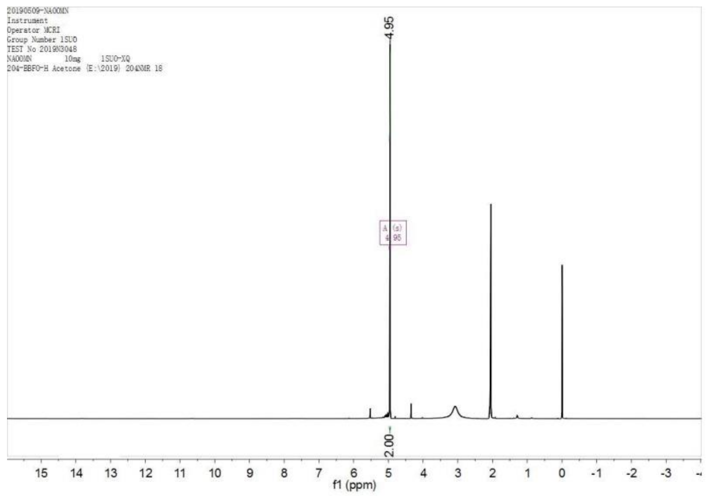

Fig. S3. ^{1}H NMR (500 MHz, Acetone-d_6) data of compound **3**.

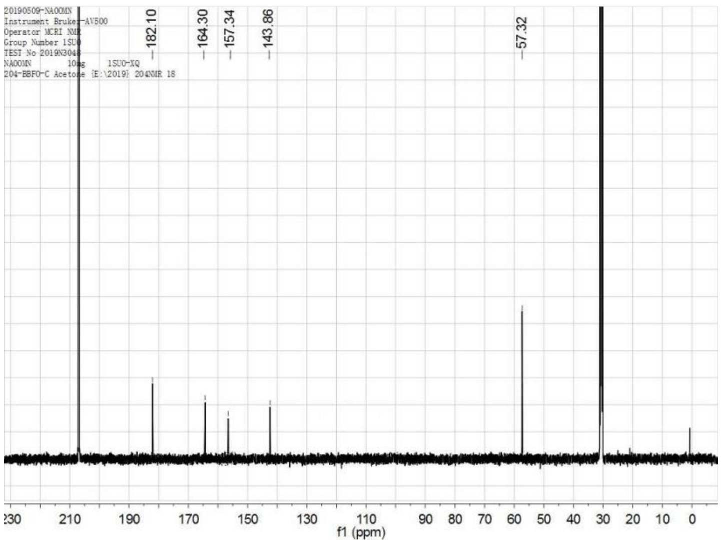

Fig. S4. ^{13}C NMR (126 MHz, Acetone-d_6) data of compound **3**.

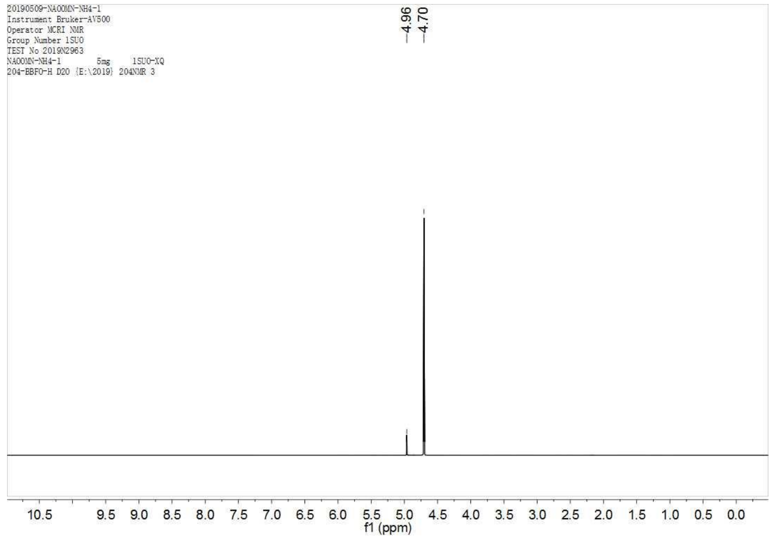

Fig. S5. ^{1}H NMR (500 MHz, D_2O-d_2) data of compound **4**.

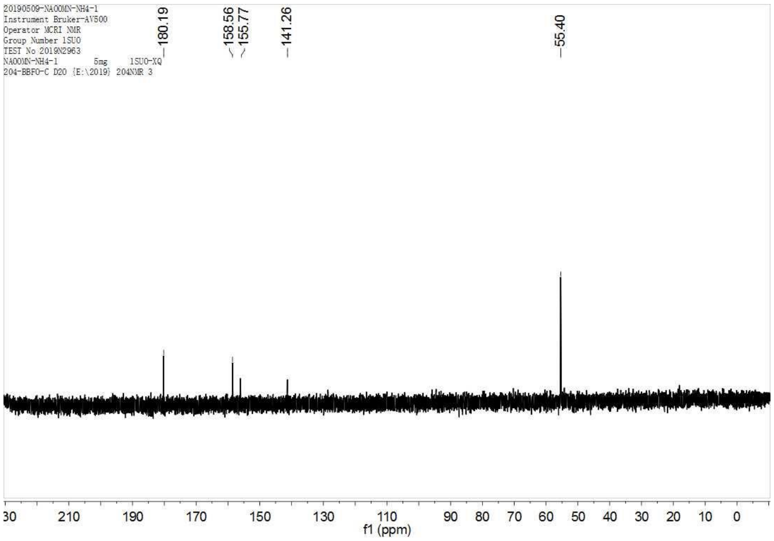

Fig. S5. ^{13}C NMR (126 MHz, D_2O-d_2) data of compound **4**.

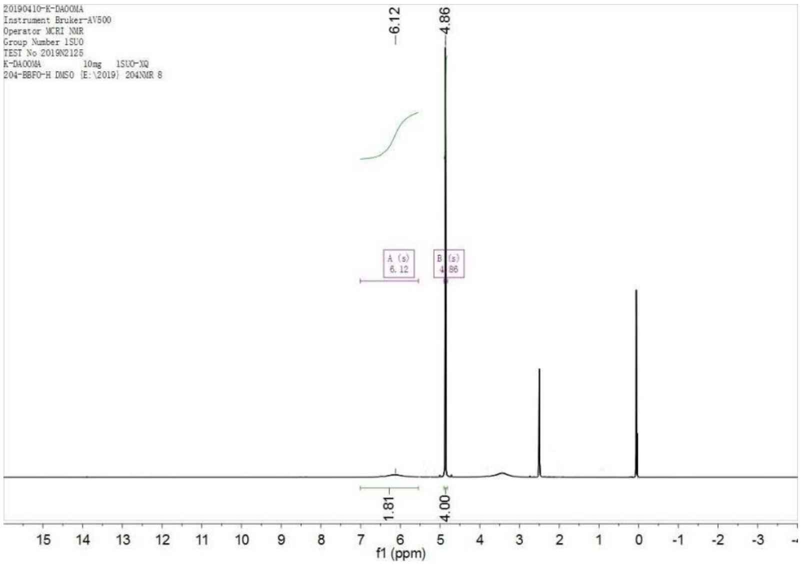

Fig. S6. ^{1}H NMR (500 MHz, DMSO-d_6) data of compound **5**.

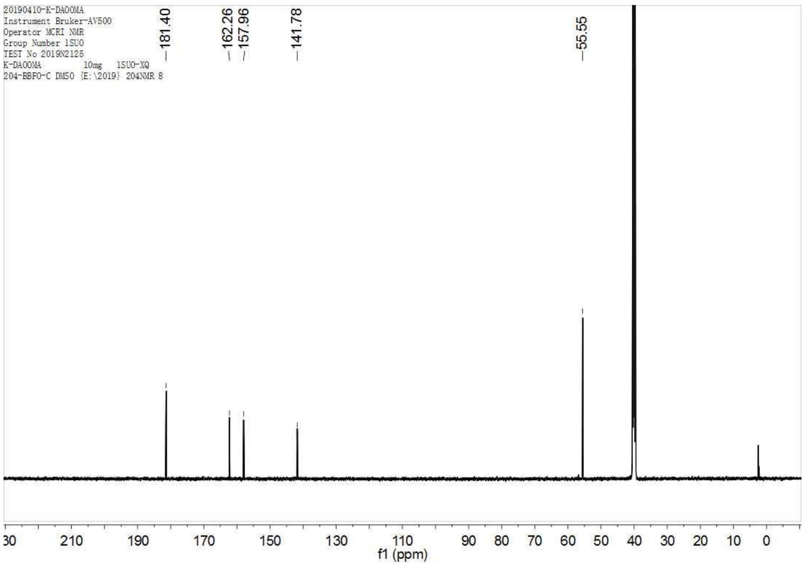

Fig. S7. ^{13}C NMR (126 MHz, DMSO-d_6) data of compound **5.**

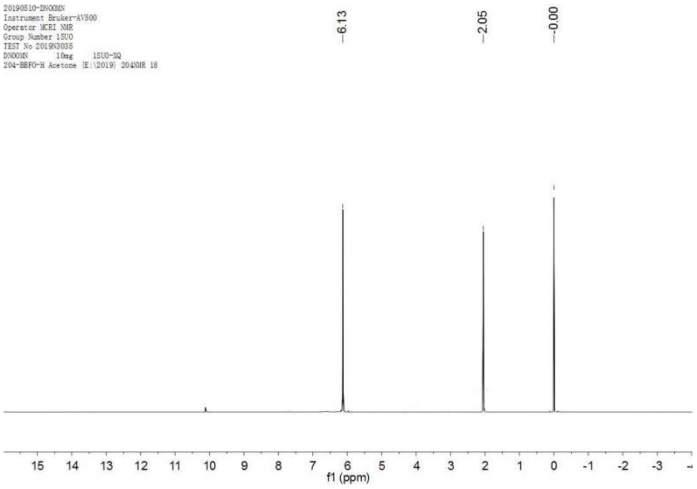

Fig. S8. ^{1}H NMR (500 MHz, Acetone-d_6) data of compound **6**.

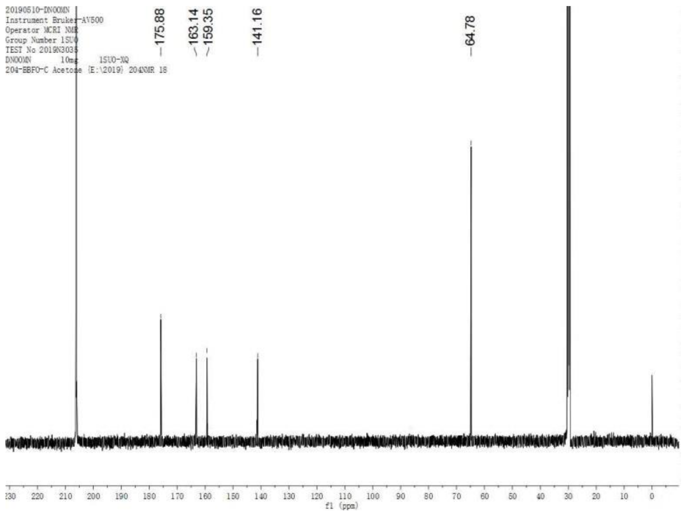

Fig. S9. ^{1}C NMR (126 MHz, Acetone-d_6) data of compound **6**.

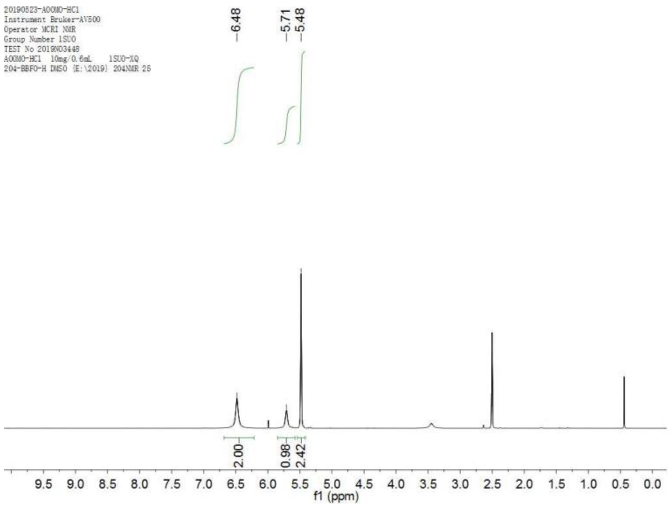

Fig. S10. ^{1}H NMR (500 MHz, DMSO-d_6) data of compound **7**

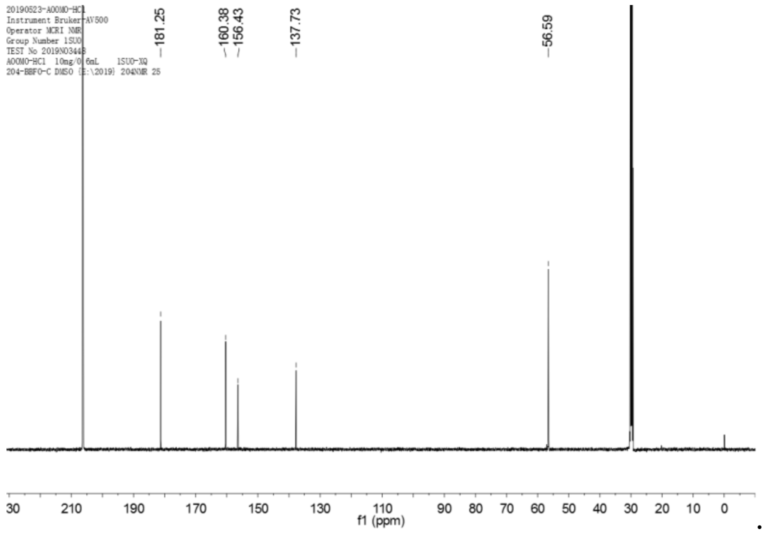

Fig. S11. ^{1}C NMR (126 MHz, DMSO-d_6) data of compound **7**.

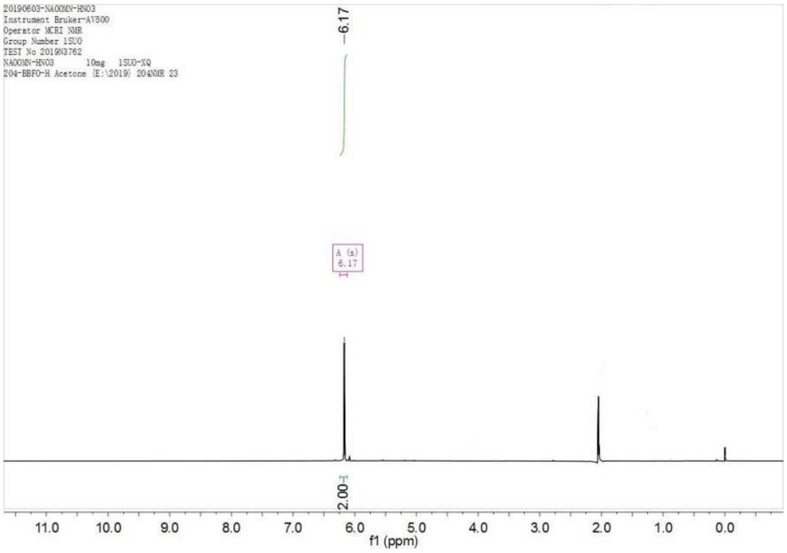

Fig. S12.^{1}H NMR (500 MHz, Acetone-d_6)compound **8**.

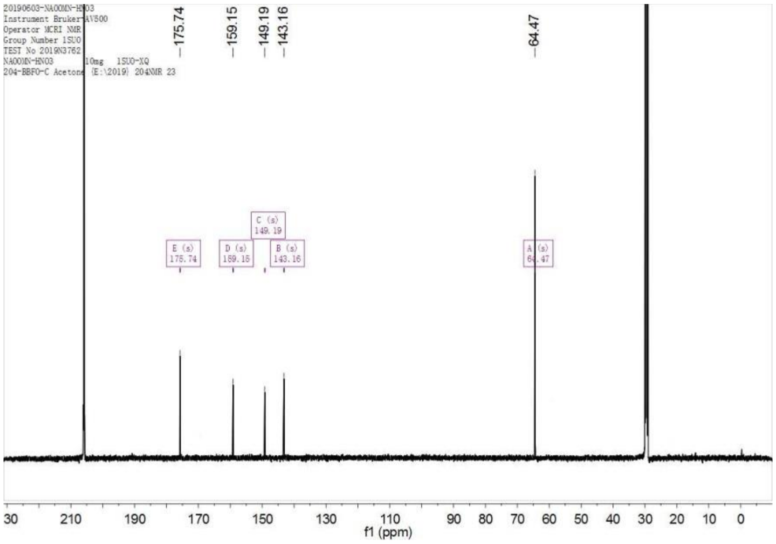

Fig. S13.^{13}C NMR (126 MHz, Acetone-d_6)compound **8**.

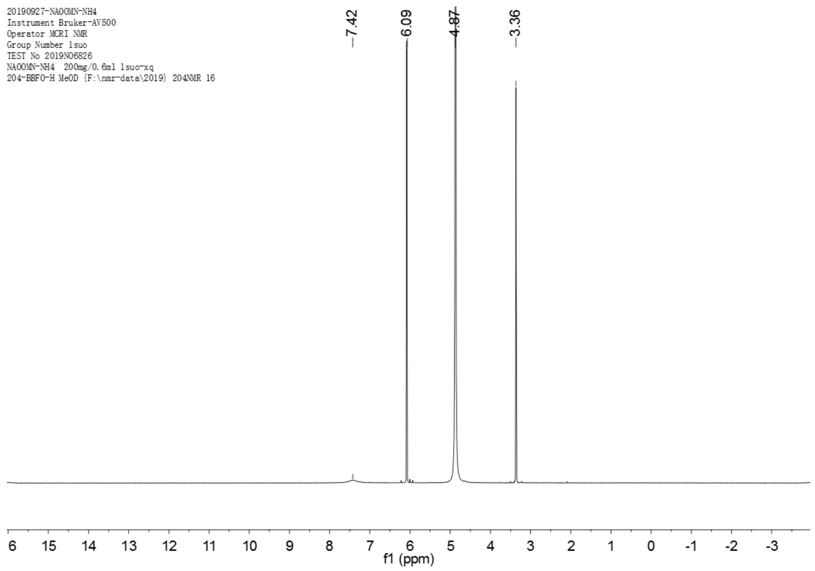

Fig. S14. ^{1}H NMR (500 MHz, CD_3OD-*d_4*)compound **10**.

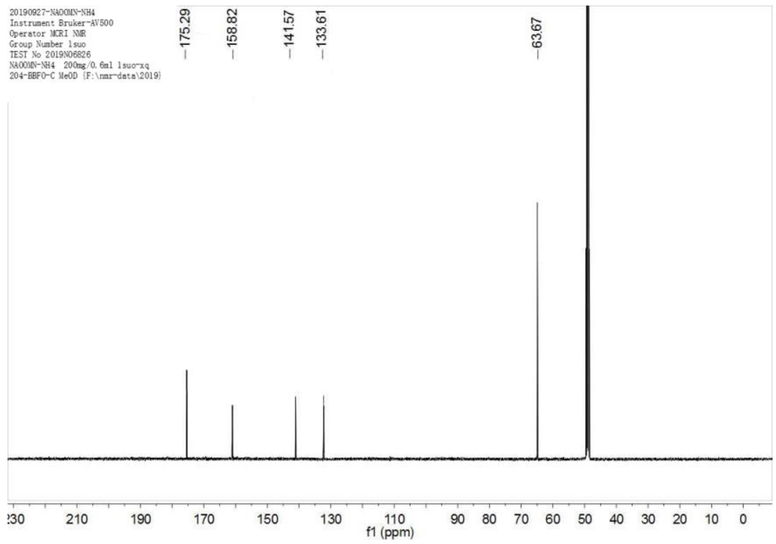

Fig. S15. ^{13}C NMR (126 MHz, CD_3OD-*d_4*)compound **10**.

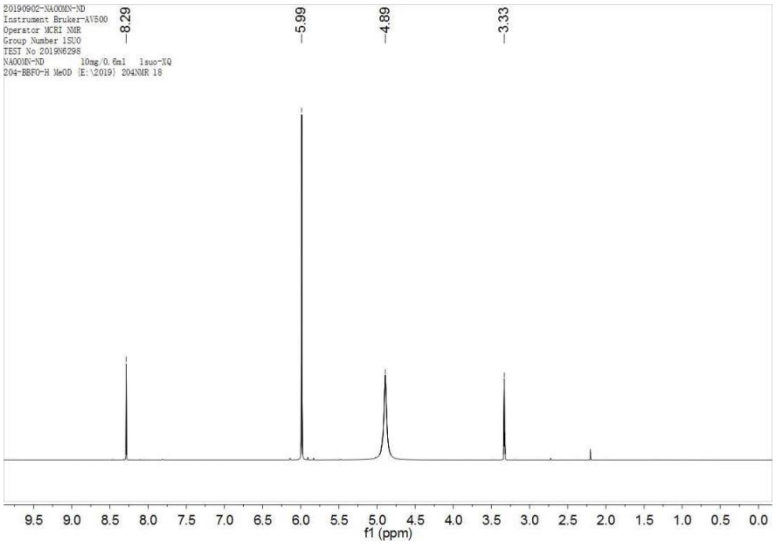

Fig. S16.^{1}H NMR (500 MHz, CD_3OD-d_4)compound **11**.

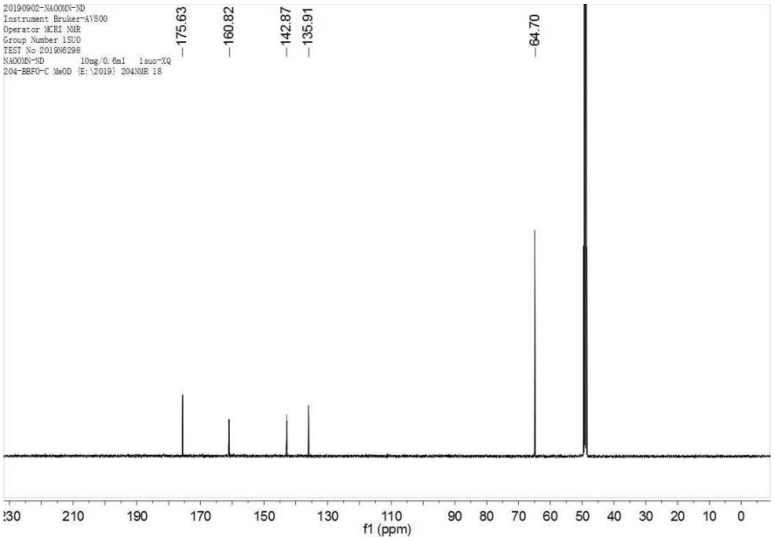

Fig. S17.^{13}C NMR (126 MHz, CD_3OD-d_4)compound **11**.

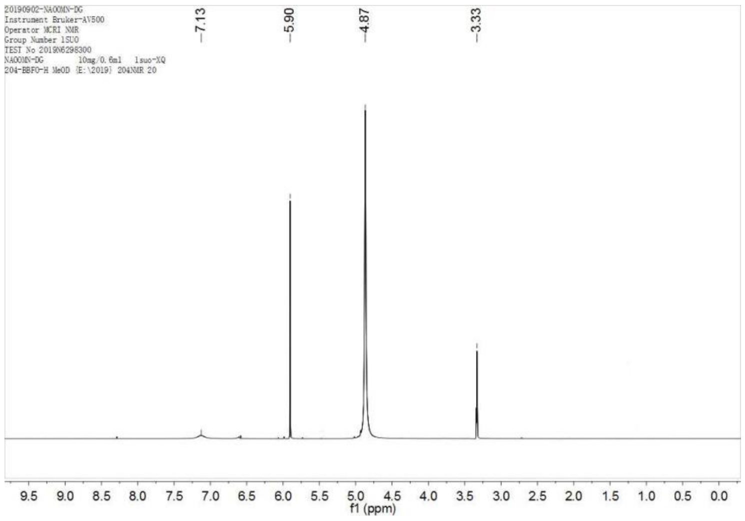

Fig. S18.^{1}H NMR (500 MHz, CD_3OD-d_4)compound **12**.

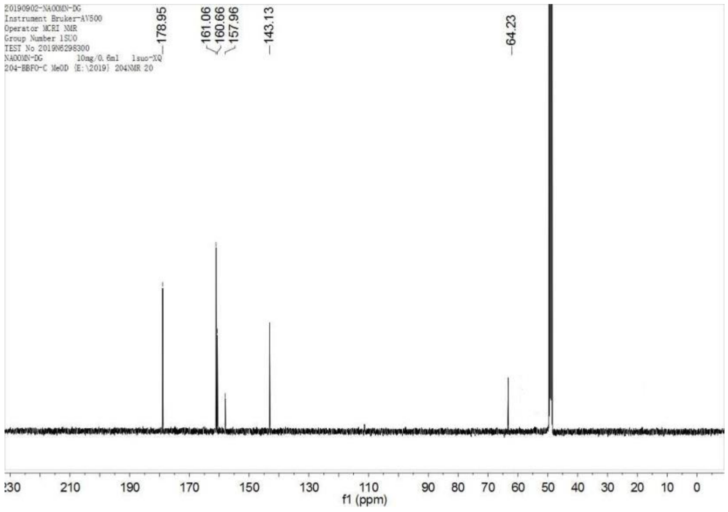

Fig. S19.^{13}C NMR (126 MHz, CD_3OD-d_4) compound **12**.

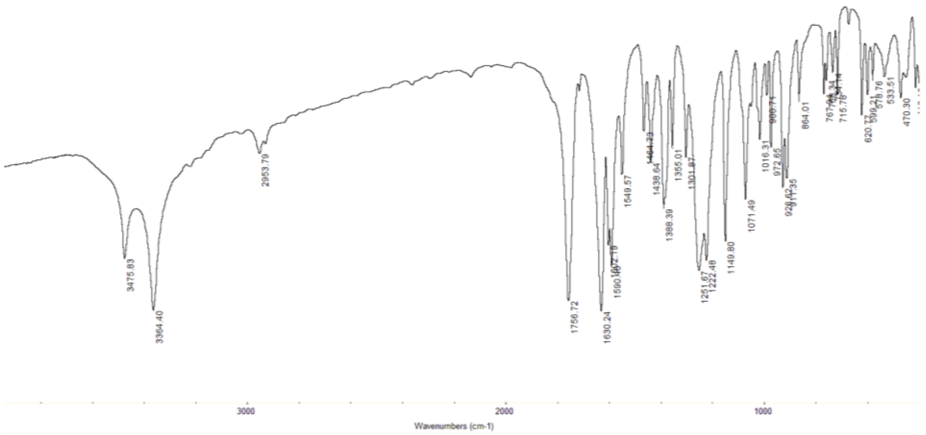

Fig. S20. IR of compound **2**.

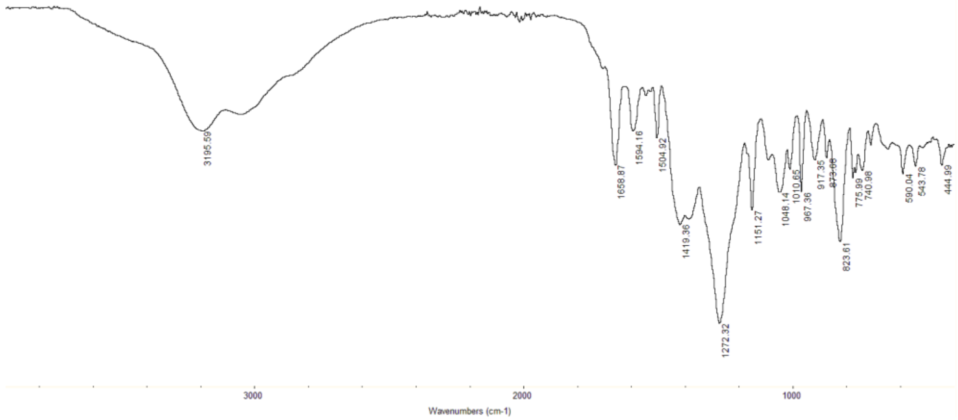

Fig. S21. IR of compound **4**.

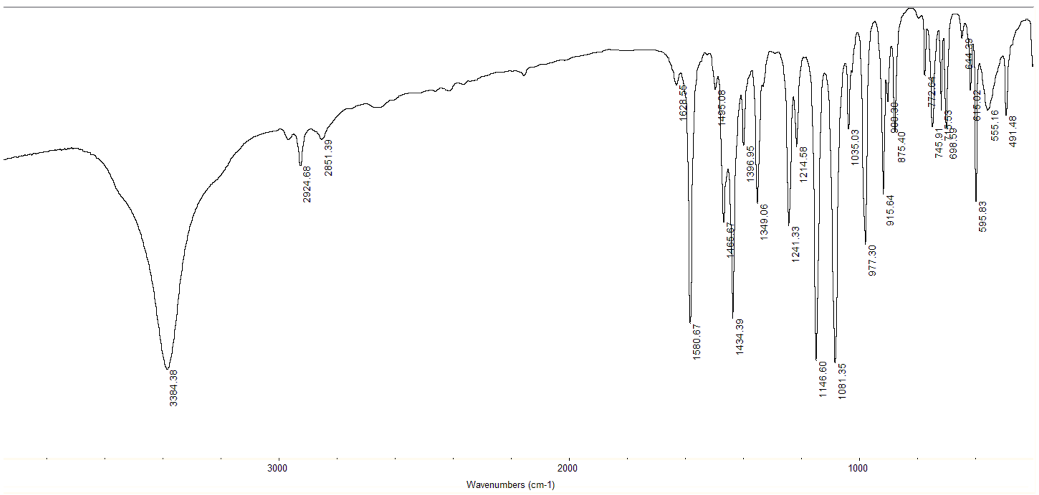

Fig. S22. IR of compound **5**.

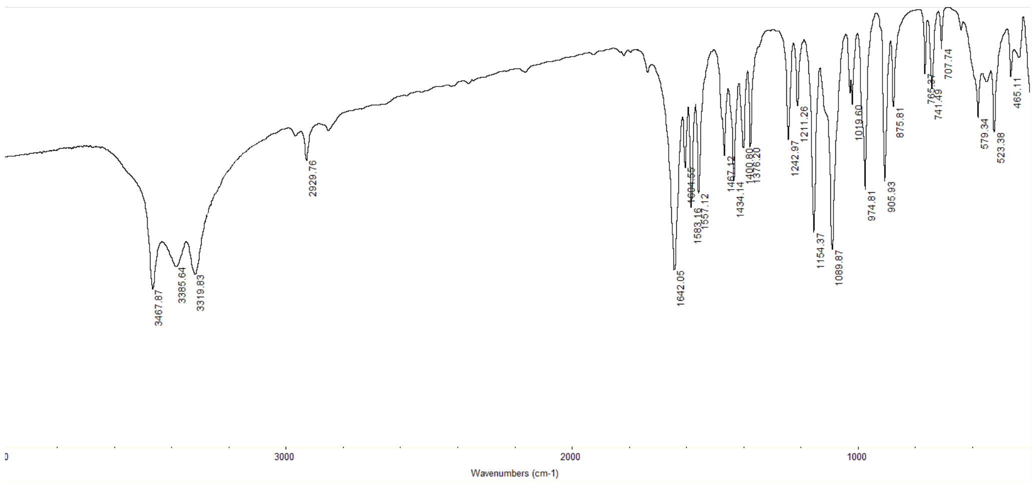

Fig. S23. IR of compound **7**.

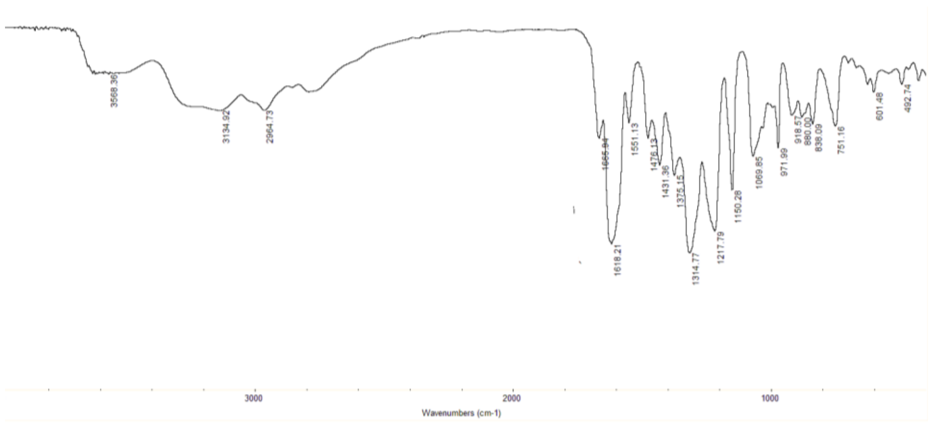

Fig. S24. IR of compound **8**.

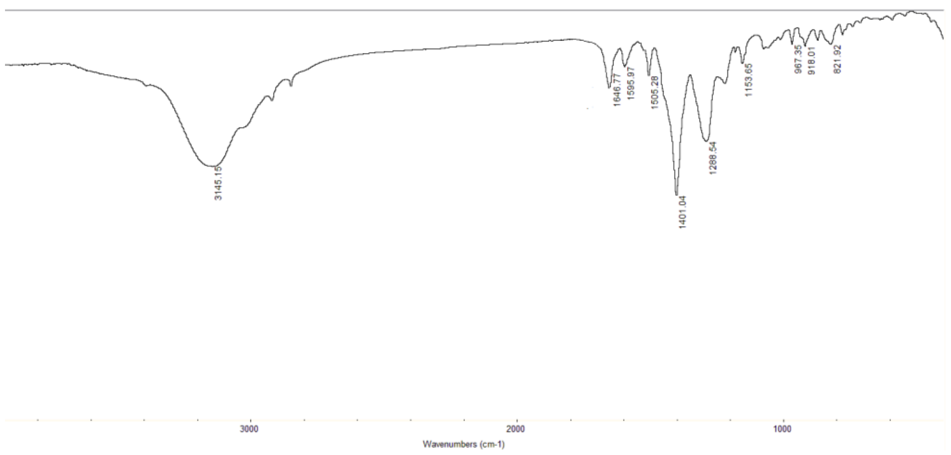

Fig. S25. IR of compound **10.**

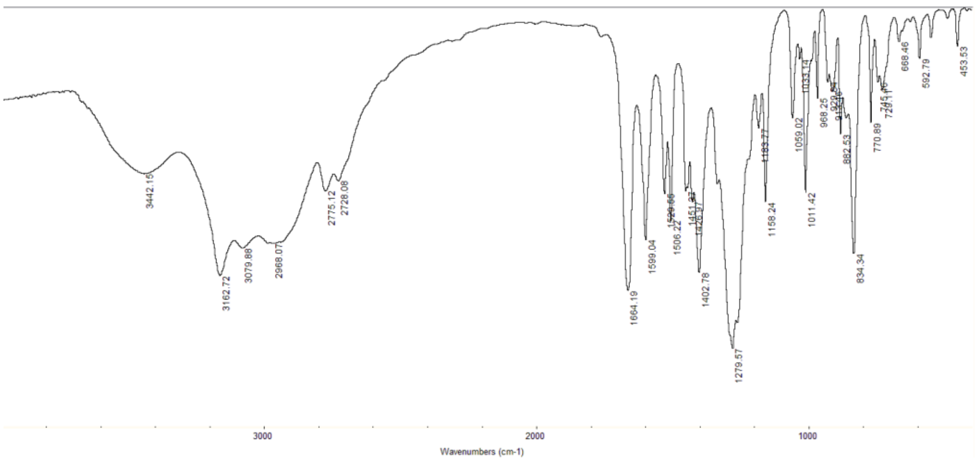

Fig. S26. IR of compound **11**.

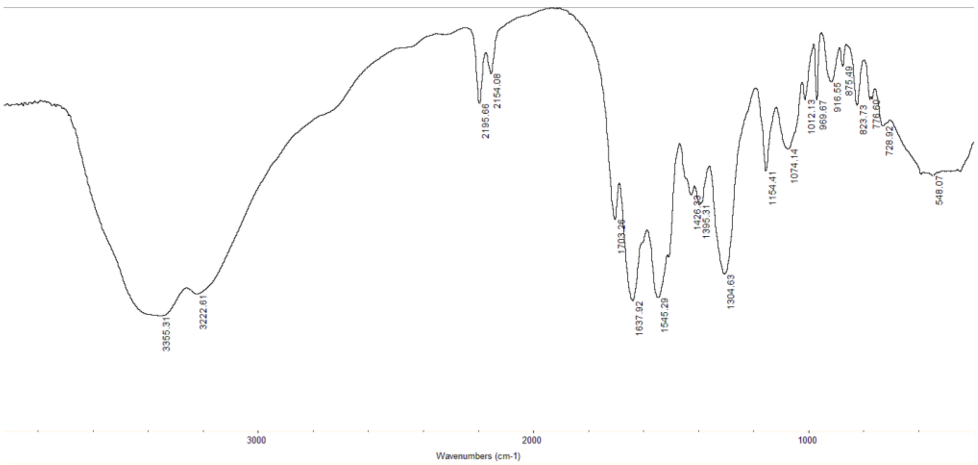

Fig. S27. IR of compound **12.**
